# Evaluate the Effectiveness of Conservative Treatment for Menorrhagia in Women who were Admitted to the Shahid Sadoughi Hospital of Yazd-Iran in 2014-2015

**Published:** 2016-09

**Authors:** M. Karimi-Zarchi, M. Zaid Abadinezhad, B. Bonyadpour, S. Kabyrpour-Ashkezar, A. M. Abhaji

**Affiliations:** 1 Department of Obstetrics and Gynecology, Shahid Sadoughi University of Medical Sciences, Yazd, Iran;; 2 Department of Midwifery, Lecturer Islamic Azad University, Shahrbabak brench, Iran;; 3 Department of Midwifery, Islamic Azad University, Shahrbabak brench, Iran;; 4 Shahid Sadoughi University of Medical Sciences, Yazd, Iran;; 5 Faculty of Pharmacy, Shahid Sadoughi University of Medical Sciences and Health Services Yazd, Iran

**Keywords:** megestrol acetat, medroxyprogesterone, GnRh agonist, Levonorgestrol IUD, endometrial ablation, menorrhagia, conservative management

## Abstract

**Background and aim:**

Abnormal uterine bleeding is one of the most common debilitating menstrual problems. The first therapeutic strategy for abnormal uterine bleeding is drug treatment. This study was, therefore, designed to determine the efficacy of megestrol, medroxyprogesterone, GnRh agonist, Levonorgestrol IUD and endometrial ablation on bleeding and also to evaluate the side effects of each methods in patients with menorrhagia who were admitted to the Shahid Sadoughi clinic of Yazd University of Medical Sciences.

**Methods and Materials:**

Based on an analytical study with consideration of patients’ medical history, 89 patients with age range of 25-50 years old were included. Each patient, under gynecologist supervision, received one of treatments for three month. The evaluation of patients bleeding in response to treatment were performed using a check list filled by patients and the results were compared before and after treatment. Medroxyprogesterone acetate, megestrol, GnRh agonist (Triptorelin embonate), Levonorgestrol IUD and endometrial ablation was used for patients as their characteristics. Each treatment was conducted for a period of 3 months. Megestrol 40 mg per day on an ongoing basis, medroxyprogesterone from 15th day of menstruation for 10 nights and Diphereline 3.75 mm (manufactured by Aria Health) were administered every 28 days. Mean of bleeding before and after treatment and complications of conservative therapy were evaluated. Statistical analysis of the data were performed using paired t test and Wilcoxon tests on spss-19 software.

**Result:**

Mean of age was 41.2 (25-50). Megestrol treatment with a frequency of 27% (24 patients) and endometrial ablation with a frequency of 20.2% (18 patients) were the most used therapy in this study. All of these ways of conservative treatment can decrease bleeding significantly. The complications of these methods of treatment were not significantly different. (*p*=0.37).Satisfaction of women after 2-3 months of treatment were increasing because spotting is common in the first months of therapy.

**Conclusion:**

The results of this study indicate that all five methods are good enough to treat menorrhagia. All these methods can replace hysterectomy, especially in this age range in which preserve fertility is of particular importance, and patients can also be protected from hysterectomy, a heavy surgery, and surgery and post surgery complications.

## INTRODUCTION

Abnormal uterine bleeding of menstruation is one of the most common debilitating problem (1) that have a significant impact on health status and quality of women’s lives (2). The prevalence of abnormal uterine bleeding is 11-13% in the general population which is increases with age and reaches 24% in women aged 36-40 years. Abnormal uterine bleeding can be acute or chronic and it is characterized by uterine bleeding with abnormal order, volume, frequency or duration in the absence of pregnancy (3). The average amount of blood loss in each period is 35 ml. Frequent bleeding with blood loss of more than 80 ml per each period will lead to anemia (4). Menorrhagia is the most common cause of iron deficiency anemia in women in which about 74.4% of anemic women have menorrhagia (5, 25, 26). The various drug treatments are, these days, available for menorrhagia that can be chosen according to the patient’s condition, including age, the need for contraception, organic causes or underlying disease, a desire to preserve fertility and the patient’s personal need (6-8). The first approach for treatment of abnormal uterine bleeding is drug therapy, such as progestins, estrogens and progesterone combination, prostaglandin inhibitors and anti Fibrinolatic (9). The aim of present study was to determine the effectiveness of non-invasive method such as megestrol, medroxyprogesterone, GnRh agonist, LNG-IUD and endometrial ablation on the amount of bleeding and the side effects of each treatment methods in patients with menorrhagia.

## MATERIALS AND METHODS

This study was a cross-sectional analytical study which was conducted on eligible women aged 25-50 years old with menorrhagia who were referred to the clinic of the shahid Sadoughi Hospital from June 2014 to 15 October 2014. Eighty-nine patients entered the study. Inclusion criteria were: 1) Age between 25-50 years; 2) Lack of uterine fibroids larger than 3 cm; 3) The absence of other pathological or iatrogenic causes for abnormal uterine bleeding; 4) Lack of systemic disease that can affect menstrual bleeding; 5) Not taking any hormonal drugs or any other medication affecting menstrual bleeding (such as Aspirin). Ultimately, each patient according to their gynecologist treated with one of the methods: medroxyprogesterone acetate, megestrol, GnRh agonist [Diphereline (Triptorelin embonate)], LNG-IUD (mirena) and endometrial ablation. Each treatment was conducted for a period of 3 months. Megestrol 40 mg per day on an ongoing basis, medroxyprogesterone from15th day of menstruation for 10 nights and ampoule of Diphereline 3.75 mm (manufactured by Aria Health) were administered every 28 days. Exclusion criteria: 1) unwillingness to continue treatment for any reason; 2) Complications that require special treatments such as anemia and pregnancy; 3) Start therapy that will interfere with treatments of this study. The evaluation of bleeding was performed by check list filled by patients and was based on the number of pads used by patients before treatment. Three months after treatment the patients’ response to treatment were evaluated again using the same check list and the results were compared before and after treatment. Statistical analyses of the data were performed using paired t test and Wilcoxon tests on spss-19 software.

## RESULTS

Megestrol treatment with a frequency of 27% (24 patients) and endometrial ablation with a frequency of 20.2% (18 patients) were the most used therapy in this study. The mean age, body mass index and number of parity in megestrol method and endometrial ablation were 41.08 and 46.9, 29.01 and 27.65, 3.27 and 2.62 respectively (Table 1).

**Table 1:** The way of treatment in the study as the age, BMI and the pregnancy

Number of pregnancy	Mean of BMI	Mean of age	percent %	The way of treat

3.27 ± 1.44	29.1 ± 3.5	41.08 ± 5.96	27%	Megestrol
2.62 ± 1.25	27.65 ± 4.2	46.9 ± 3.7	20.2%	Endometrial ablation
3.7 ± 2.31	29.23 ± 4.9	38.5 ± 6.04	18%	IUD mirena
2.37 ± 1.54	28.1 ± 5.4	38.3 ± 9.3	18%	Medroxyprogesterone acetate
4.7 ± 2.7	28.14 ± 3.3	40.6 ± 7.7	16.9%	Diphereline

The mean scores before and after treatment show that the average of all therapeutic methods used in this study significantly increased after the intervention (α≤0.001). So the amount of bleeding after treatment with any of the 5 treatment significantly decreased (Table 2).

**Table 2:** Mean of bleeding before and after of treatment

The significance level	Mean	Variable	

<0.001	36.1 ± 96.6	before	Megestrol
	09.2 ± 17.2	after	
<0.001	78.0 ± 83.7	before	Endometrial ablation
	28.2 ± 94.1	after	
<0.001	3.1 ± 56.7	before	IUD mirena
	57.1 ± 75.1	after	
<0.001	5.1 ± 56.6	before	Medroxyprogesterone acetate
	47.2 ± 56.2	after	
<0.001	64.1 ± 7	before	Diphereline
	37.2 ± 8.2	after	

According to Table 3, there were no complications in any of the treatments. The most common complication was spotting which was more common in the group who were treated with megestrol (10 patients).

**Table 3:** The complication rate in groups of study

The other	weight gain	Nause avomiting	headache	spotting	Without complication	The way of treatment

-	-	-	1 (6.2%)	10 (41.7%)	13 (54.2%)	Megestrol
-	-	-	-	3 (16.7%)	15 (83%)	Endometrial ablation
-	-	-	-	6 (37.5%)	10 (62.5%)	IUD mirena
-	-	1 (6.2%)	1 (6.2%)	4 (25%)	10 (62.5%)	Medroxyprogesterone acetate
1 (6.2%)	-	-	-	8 (53.3%)	6 (40%)	Diphereline

Other complications refers to: abdominal distention and whole body pain.

## DISCUSSION AND CONCLUSION

Abnormal uterine bleeding is the single most common complaint of women of reproductive age with which they go to the doctor (10). As studies show, heavy menstrual bleeding is the most common cause of visits to the doctors and leave from work among women of reproductive age (11). The results of this study demonstrated a significant differences between the amount of menstrual bleeding before and after treatment with Megestrol (*P*>0.001), as the bleeding was reduced after treatment. Muneyyirci-Delale study (12) conducted in New York, revealed that treatment with megestrol decreases the mean endometrial thickness to 14 mm and also reduces the number of bleeding days from 54 days to 3 days. Williams (13) who evaluated the impact of megestrol on 21 patients with menorrhagia also observed that 18 patients recovered after treatment. The most common complication in their study was spotting that were consistent with the results of this study.

Based on the results of this study, the group treated with endometrial ablation demonstrated a significant differences in bleeding before and after the intervention (*P*>0.001). This result is similar to findings of Bouzari (14), which was performed on 30 patients with menorrhagia in Babol. According to our results, the amount of bleeding in endometrial ablation procedure after 3, 6 and 12 months was reduced to 36.7% 43/3% and 36.7% respectively. In the study of Mansouri (15) also 2 to 3 months after treatment a significant reduction in menstrual bleeding has been reported in which none of the patients needed subsequent hysterectomy. Penezic study obtained similar results (16). Waddell in Canada (17) also examined the effects of uterine ablation in reducing symptoms of premenstrual. Their results indicated that premenstrual symptoms (anxiety, depression, headaches, breast swelling and tenderness) one year after the destruction of the endometrium was significantly reduced (*P*>0.0250) was dropped. In this study, the majority of patients (83%) did not report complications after ablation and only 3% had spotting. As the findings of this study show, a group that had been treated with LNG- IUD (mirena), after IUD insertion demonstrated a significant difference in the amount of menstrual bleeding than before. Dhaman Gaonkar (18) investigated the impact of this type of IUD on abnormal uterine bleeding and reviewed the results obtained after 4 months, one year and two years after treatment and observed that bleeding between menstrual periods of patients had been decreased 80%, 95% and 100 % respectively. Also 4 months after treatment 7.8% increase was observed in patients’ hemoglobin. Rcepe reviews (19) showed that in the majority of patients the amount of bleeding was reduced and spotting was the only complication in which it was consistent with this study. Karimi Zarchi (20) also showed that abnormal uterine bleeding were significantly reduced after treatment with levonorgestrel IUD (*P*>0.047). Began, Long and El Behery (21-23) achieved similar results as well.

According to the results of this study, treatment with medroxyprogesterone acetate in women with uterine bleeding is significantly effective in reducing the amount of blood loss (*P*>0.001). Mokhah (24) showed that treatment with medroxyprogesterone acetate for 3 menstrual cycles caused a significant reduction in the amount of bleeding (*P*>0.0001). Spotting in their study was the most common complication reported which is consistent with our results. In a study conducted by Karim Zarchi (20), it was found that side effects caused by treatment with medroxyprogesterone was significantly higher than the IUD-LNG (*P*>0.003).

The results of this study showed that GnRh agonists can be effective in treatment of severe uterine bleeding. Ouladsahebmadarek review (9) on 250 women ages 40 to 55 years old with abnormal uterine bleeding which was carried out in Iran, was found that bleeding in 91.8% of patients was stopped after 2 doses of Diphereline and complications such as mild back pain, frequent urination and depression were significantly higher in patients with hysterectomy, (*P*>0.03, *P*<0.03, and *P*<0.001).

As the results of this study suggest all five methods evaluated in this study are suitable to treat menorrhagia. The first line of therapy in a person with abnormal uterine bleeding and endometrial hyperplasia without atypia is drug treatment. They respond well to oral therapy with progestin, but on the other hand, low acceptance of patients and systemic side effects may decrease the effectiveness of treatment. LNG-IUD, without the disadvantages of oral progestins, can be successful in the treatment of hyperplasia. Even in cases of failure to respond to treatment, conserving surgery such as endometrial ablation is recommended.

All these methods can replace hysterectomy, especially in this age range in which preserve fertility is of particular importance, and patients can also be protected from hysterectomy, a heavy surgery, and its complications.
